# Successful use of endovascular aneurysm repair in the treatment of acute aortoduodenal syndrome

**DOI:** 10.1093/jscr/rjac372

**Published:** 2022-08-16

**Authors:** Carolina A Orsi, Hishaam Ismael, David B Kerns

**Affiliations:** Department of General Surgery, University of Texas Health Science Center, UT Health East Texas, Tyler, TX, USA; Department of Surgery, Division of General Surgery/Surgical Oncology/Hepatobiliary Surgery, University of Texas Health Science Center, UT Health East Texas, Tyler, TX, USA; Department of Surgery, Division of Vascular Surgery, University of Texas Health Science Center, UT Health East Texas, Tyler, TX, USA

## Abstract

Aortoduodenal syndrome is a rare phenomenon, described as a duodenal obstruction secondary to direct compression from an abdominal aortic aneurysm. Although traditionally managed with gastrointestinal bypass and subsequent open aortic aneurysm repair, such procedures hold high mortality. Therefore, there is great value in treating this syndrome with an endovascular, minimally invasive technique that is both consistent and successful. This case report recounts how an endovascular aneurysm repair successfully treated a patient with acute aortoduodenal syndrome. No postoperative complications occurred and the duodenal obstruction was relieved without the need for surgical re-intervention. This conveys the advantageous utility of endovascular aneurysm repair in treating patients with aortoduodenal syndrome.

## INTRODUCTION

Aortoduodenal syndrome (ADS) is a known, yet rare, phenomenon described as a mechanical obstruction of the duodenum secondary to direct compression from an abdominal aortic aneurysm (AAA) [1]. This was first explained by William Osler and has only been reported about 40 times within the literature since [2]. Patients with ADS most commonly present with vomiting, abdominal pain, electrolyte disturbances and often a pulsatile abdominal mass [3]. The diagnosis is confirmed via contrast computed tomography (CT), followed by upper gastrointestinal contrast-enhanced imaging to rule out other potential causes of duodenal obstruction [4].

Management of ADS ranges from conservative therapy to surgical decompression [4, 5]. Traditionally, treatment involved relieving the mechanical bowel obstruction first with gastrointestinal bypass, followed by open aortic graft placement [5]. Unfortunately, attempts at gastrointestinal bypass prior to aortic surgery have high failure rates [6]. The first endovascular approach to treat ADS was described by Newman et al. in 2015, marking the novel technique as viable and effective [7]. Recent reports continue to reveal how endovascular aneurysm repair (EVAR) serves as a consistent treatment option for those with ADS [8].

Here, we report a case where EVAR successfully treated ADS without any postoperative complications or bypass procedures. Although the best method of therapeutic intervention for ADS has yet to be established, this case illustrates how EVAR can be utilized as a safe and incredibly effective technique in relieving duodenal obstruction caused by ADS.

## CASE REPORT

This patient is a 65-year-old male with previously diagnosed AAA who presented to the emergency department due to acute onset of abdominal distension and cramping. He also experienced multiple episodes of nausea, vomiting, and hiccups. On exam, the patient was tachycardic (101 bpm), but normotensive (110/68 mmHg), and in no acute distress. There was a pulsating mass at the epigastrium, without signs of peritonitis. CT scan revealed an infra-renal AAA measuring 6.7 cm in diameter with an interval increase in size since last seen 4 years prior (5.1 cm diameter). The stomach and proximal duodenum were markedly distended with an abrupt change in caliber of the duodenum as it coursed anterior to the AAA.

**Figure 1:**
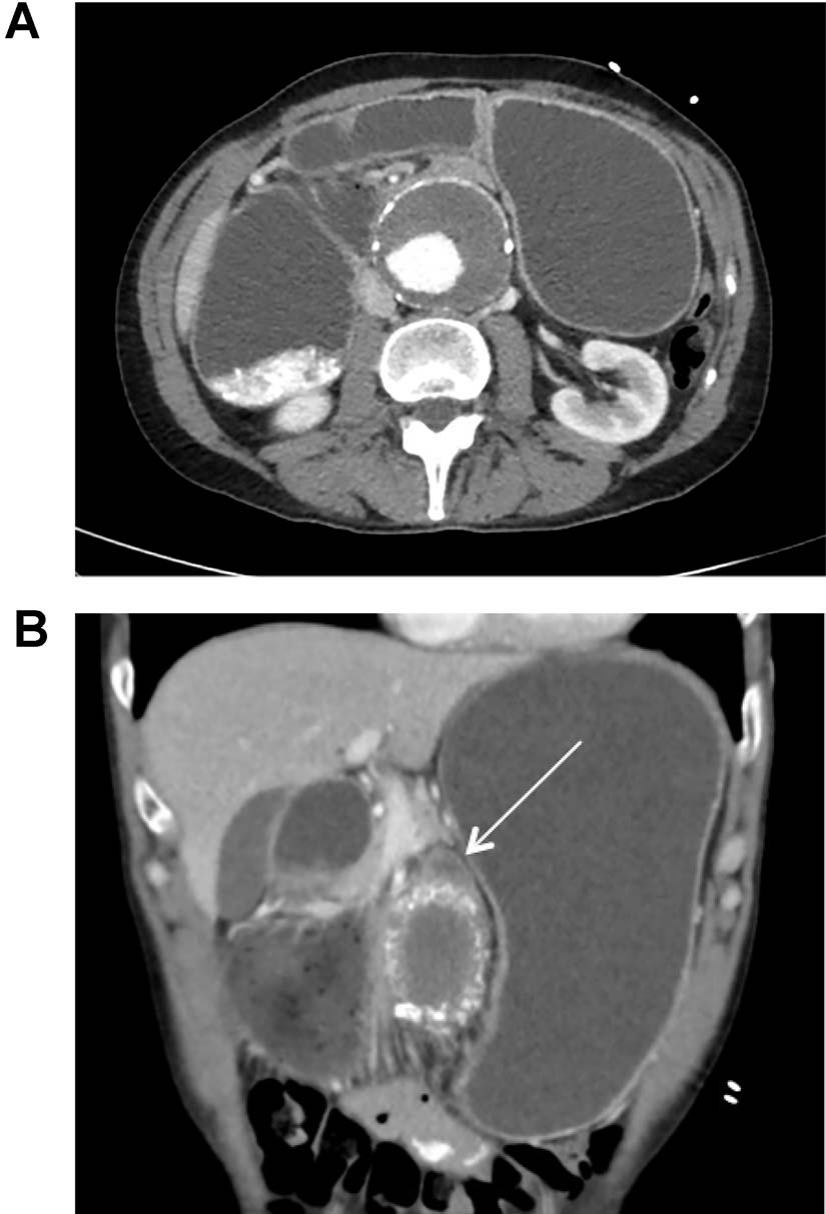
CT scan illustrating duodenal obstruction secondary to external compression of abdominal aortic aneurysm (**A**); CT scan illustrating transition point of obstructed duodenum as it traverses the abdominal aortic aneurysm (**B**, arrow).

The patient was initially treated with nasogastric decompression, intravenous fluid resuscitation, and antiplatelet therapy (Aspirin/Plavix). CT Angiogram was then obtained for further evaluation of the patient’s vascular anatomy (Fig. 1). Additionally, the patient underwent esophagogastroduodenoscopy to rule out other etiologies for duodenal obstruction, during which none were found. 48 hours later, he was taken to the operating room for EVAR.

**Figure 2:**
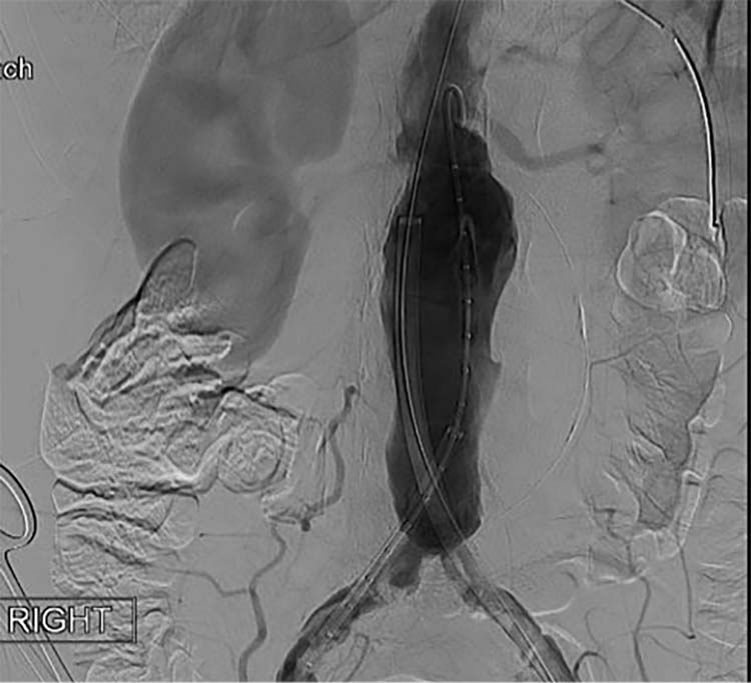
Intraoperative fluoroscopy illustrating infra-renal, abdominal aortic aneurysm prior to graft placement.

**Figure 3:**
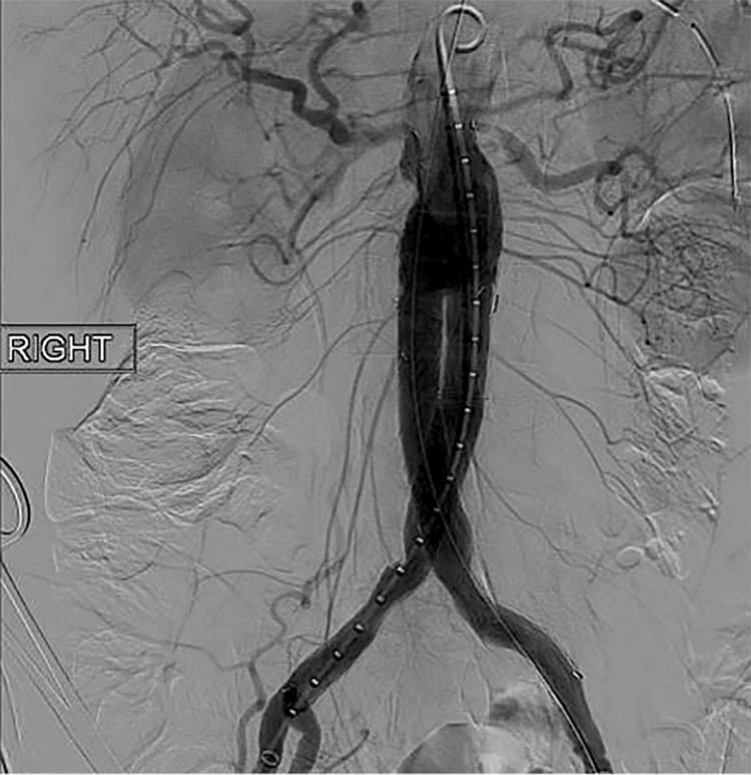
Intraoperative fluoroscopy illustrating successful endovascular aneurysm repair (EVAR).

To obtain vascular access, bilateral cutdown technique was used to dissect the common femoral arteries. The femoral arteries were cannulated, the patient heparinized, and an arteriogram obtained (Fig. 2). The main body of the graft was deployed without complication, followed by bilateral iliac limb extensions. The graft was treated with the Molding & Occlusion Balloon (MOB) at all attachment points. Repeat arteriogram demonstrated excellent technical result without evidence of endoleak (Fig. 3). Protamine was administered for reversal of heparin, bilateral groin incisions were closed, and nasogastric tube (NGT) was left in place on low intermittent wall suction. Aspirin/Plavix therapy was continued.

The patient had an uncomplicated postoperative course. On postoperative day (POD) 2, the patient began to have bowel movements and tolerated NGT clamping. Total parenteral nutrition was initiated in anticipation that the patient would be unable to tolerate oral nutrition, but this was discontinued on POD 3. The NGT was removed, a clear liquid diet started, and a small bowel follow through was performed on POD 3 that revealed no further obstruction. The patient was slowly advanced to a regular diet and subsequently discharged on POD 5.

## DISCUSSION

ADS is quite rare and its therapeutic management remains controversial as new techniques evolve in practice [1]. Many cases now reveal how advantageous endovascular interventions of ADS can be versus the traditional gastrointestinal bypass and open repair [1, 5]. This case serves as a favorable example of utilizing EVAR in treating ADS through minimally invasive techniques, without postoperative complications, and without need for surgical re-intervention.

We attribute the reduction of aneurysmal sac pressures and wall tensions after endovascular aortic graft placement to explain the pathophysiology behind why EVAR relieves duodenal obstruction in ADS [9]. A report by Sonesson et al. describes that aneurysmal sac pressures can become as low as 17 mmHg in as little as 6 hours after graft placement [10]. With decreasing sac pressure, decreasing wall tension and potential shrinking of the aneurysmal diameter, external compression upon the duodenum lessens enough to reestablish bowel patency and allow the patient to tolerate oral intake.

There are several reports regarding ADS that suggest the condition is only associated with average aneurysm diameters of 78 mm [11]. However, Ahn et al. and Esposito et al. both confirm and emphasize that ADS can occur even with aortic aneurysms as small as 50 mm [5, 12]. This correlates with our patient, as his aneurysm was 67 mm in diameter while still presenting with severe duodenal obstruction.

Similar to ADS, the duodenum can become entrapped between a normal aorta and the superior mesenteric artery (SMA) in SMA syndrome due to a narrowed aortomesenteric angle [13]. Although most commonly associated with rapid weight loss, it is described by Baltazar et al. to also occur in other instances [14]. It is often presumed that ADS and SMA syndrome are independent entities, as their obstructive mechanisms stem from different etiologies within the abdomen [15]. However, seeing as both narrowing of the aortomesenteric angle could exist in concordance with an AAA, it is reasonable to postulate that both conditions could present simultaneously [13]. SMA syndrome was considered in this case; however, the extent the SMA played in the clinical course was uncertain.

As described earlier, open aneurysm repair and gastrointestinal bypass in those with ADS holds significant mortality [9]. Thus, there is great utility in treating this syndrome with minimally invasive techniques that are both consistent and successful. EVAR allowed our patient to avoid open surgical interventions and discharge in stable condition while tolerating oral intake. Therefore, this case affirms the safe and effective nature of EVAR for initial management and definitive treatment of ADS without complications or setbacks.
